# Pain modulation in the spinal cord

**DOI:** 10.3389/fpain.2022.984042

**Published:** 2022-09-13

**Authors:** Clifford J. Woolf

**Affiliations:** FM Kirby Neurobiology Center, Boston Children's Hospital and Department of Neurobiology, Harvard Medical School, Boston, MA, United States

**Keywords:** pain, spinal cord, gate control theory, inhibition, presynaptic

## Abstract

The sensory inflow from the periphery that triggers innocuous and painful sensations is highly complex, capturing key elements of the nature of any stimulus, its location, intensity, and duration, and converting this to dynamic action potential firing across a wide population of afferents. While sensory afferents are highly specialized to detect these features, their input to the spinal cord also triggers active processing and modulation there which determines its output, to drive the sensory percept experienced and behavioral responses. Focus on such active spinal modulation was arguably first introduced by Melzack and Wall in their Spinal Cord Gate Control theory. This theory has had a profound influence on our understanding of pain, and especially its processing, as well as leading directly to the development of clinical interventions, and its historical importance certainly needs to be fully recognized. However, the enormous progress we are making in the understanding of the function of the somatosensory system, means that it is time to incorporate these newly discovered features into a more complex and accurate model of spinal sensory modulation.

## Introduction

It is now almost six decades since the Gate Control Theory was first articulated in a major review in Science by Melzack and Wall (1). The article has been cited almost 16,000 times and has had a truly profound impact on our approach to the understanding and treatment of pain (2–6). However, while the Gate Control Theory offered new insight into the potential mechanisms responsible for the operation and modulation of the somatosensory system, and certainly triggered much work on nociceptive circuit organization in the spinal cord, it was based on a very limited data set. Because the neuroscience field has advanced technically in ways that would have been unimaginable in 1965, it is time now to recognize the over-simplistic notions that constituted the basis for the original gate control theory, something Pat Wall himself recognized in an article in 1996 (7), and begin to replace it with an up-to-date assessment of spinal nociceptive processing, one that more fully captures our current understanding of the operation of the spinal cord in the generation of pain.

A major driver of the original Gate Control theory was to address how neurons encode the signals leading to the sensory experience of pain. At that time there was a vigorous debate as to whether somatic sensations resulted only from a specific set of highly specialized sensory neurons triggering activity in clearly delineated circuits or was the consequence instead of the spatiotemporal patterns of activity in non-specialized afferents. Melzack and Wall explicitly stated that they considered both these theories to be both partially wrong and partially right. The knowledge on the specialization and nature of different sensory afferents was at that time limited, especially for C-fiber neurons, nevertheless the specificity theory pushed for sensation being a fixed and direct path (labeled line) from a defined peripheral trigger that only activated a specific set of specialized sensory neurons leading to the activation of those brain circuits that drive the appropriate sensory perception. In contrast, the pattern theory held that there was no modality specificity, it was only the nature of the complex spatiotemporal patterns of activity in a broad set of unspecialized neurons that created a central pattern of activity that could either lead to innocuous or painful sensations. Melzack and Wall came up with a new theory to overcome the weaknesses they identified in both these theories, a model which focused primarily on a balanced central control (or gate as they phrased it, following the term used then for the “gating” role of transistors in electrical circuits) of afferent input into the spinal cord. They hypothesized that activity in A fibers would close a spinal gate by activating an inhibitory interneuron in the substantia gelatinosa, which would then presynaptically block input from all slowly conducting unmyelinated C fiber to spinal cord-to-brain projection neurons. In contrast, activity in C-fibers would shut off the activity of the inhibitory interneuron in the dorsal horn, opening the gate and driving activation of the neurons that project from the spinal cord to the brain to produce pain. The gate control theory only had a very limited set of components. Input was either in low threshold A fibers or high threshold C-fibers, and these A and C fiber inputs were proposed to feed into only two sets of neurons in the spinal cord, a projection neuron (which they called a Transmission cell which received excitatory input from both A and C fibers (low and high threshold input), and a single class of inhibitory interneuron in the superficial dorsal horn which they hypothesized was only activated by A fibers and inhibited by C fibers, and that only had presynaptic inhibitory synapses, which was the proposed primary mechanism for the gate control—shutting off sensory inflow in C-fibers to the spinal cord to reduce pain or enabling such inflow to produce pain. In addition, they proposed that there was a central inhibitory input from the brain to the spinal circuit but provided minimal details on the origin or nature of this control.

The reason we need to move on from the original gate control theory is that the model, while inspiring and of great historical importance, is we must now recognize, outdated and too simplistic. We now know there are both low threshold C-fibers (low threshold C mechanoreceptors) and high threshold Aβ and Aδ fiber nociceptors (7), so pain is not simply a consequence of the balance of activity between A and C fibers, or of the control of the input of these afferents into the spinal cord. Primary sensory neurons are highly specialized to detect specific aspects of stimuli from the organs they innervate, as revealed by the Nobel Prize to David Julius and Ardem Patapoutian in 2021 for identifying the ion channel transducers of many external stimuli (8). Primary afferents are all excitatory in any case, so it is not clear how C fiber input could directly inhibit inhibitory interneurons, as proposed in the gate control theory. Furthermore, the theory required some low level of constitutive ongoing activity in C fibers to hold the gate open, but there is minimal if any such activity, except in disease states. In addition, most projection neurons in the dorsal horn are nociceptive-specific, not wide dynamic range, as specified in the Gate Control model, and there is not just one type of projection/transmission neuron but several distinct types with different inputs and projecting to different nuclei in the brainstem or brain. There are also very many different specific types of inhibitory and excitatory interneurons in the dorsal horn.

The circuitry of the spinal cord is, therefore, very complex, and we need to recognize and understand this. Indeed, while presynaptic inhibition does occur, it is we now appreciate, less of a controller than postsynaptic inhibition, and that both inhibition and excitation have multiple different feed forward and feedback elements (9–12). Finally, in addition to the expanded understanding of the nature and dynamics of the sensory inflow generated in highly specialized sensory neurons and the multiple different circuits they activate in the dorsal horn, we also now recognize that non-neuronal cells (glia and microglia) have an active role in generating pain-triggering processes in the spinal cord (13, 14), something not featured in the gate control theory. In essence, the nature of the sensory inflow, the complex circuits they activate as well as the output from the spinal cord, are all much more complex and differently organized from the model spelt out in the Gate Control theory.

One of the most novel features of the original theory was that it highlighted the presence of, and opportunity for manipulation of the gate control circuit, with primary focus on how activity in A fibers would shut the gate by reducing the inflow of the C-fiber input to the circuit. This was despite the fact that tactile allodynia is a major feature of clinical pain, where activity in low threshold A fibers, which normal evokes innocuous sensations, now produces pain. The presence of tactile allodynia is the precise opposite of the major predicted element of the gate control theory, that A fiber input closes the gate. Another issue is secondary hyperalgesia, how a lesion triggers changes in sensitivity outside the area of the lesion, for which no clear explanation was provided in the proposed gate control mechanisms, even though it is a common feature of clinical pain hypersensitivity.

The major therapeutic implication of the spinal gate control theory was that an increase in A fiber input, by decreasing C fiber input, would be analgesic, and this proposal contributed to the development of neuromodulation for pain relief, either through transcutaneous nerve stimulation or spinal cord stimulation. While this remains one of the major positive consequences of the model, and multiple series of interventions designed specifically to close the gate that have been successfully used in many thousands of patients, we need to recognize that for many contemporary neuromodulation approaches, the most effective analgesia is generated at a high frequency with no paresthesia (15–17) and that this points to the analgesia as not simply being due to driving normal levels of A fiber input into the spinal cord, something also reinforced by the observation that neuromodulation analgesia is much more widespread than a model of a balanced input from spatially restricted areas would predict (18).

Certainly, a balance of excitation and inhibition, as in all parts of the CNS, plays a critical role in nociceptive circuit function, but this is much more than just a presynaptic filtering of afferent input. Both the loss of inhibitory interneuron activity (disinhibition) and an increase in the excitability/synaptic input of projection neurons (central sensitization) are major contributors to the generation of acute and many clinical pain syndromes, whereas loss of A fiber input is simply not a major player. The theory would predict that a loss only of A fibers would produce spontaneous pain, but such a selective loss of A fibers is a rare event and appears not to cause pain while microneurography in patients reveals that spontaneous pain is largely driven through activation of nociceptors (19), suggesting that “loss of gating” alone may not initiate pain.

What progress have we made in our study of sensory processing and its modulation in the spinal cord since 1965? The opportunities provided by the selective optical activation or inhibition of particular primary afferents or of distinct CNS neuronal populations (20–24), combined with chemogenetic manipulations, particularly DREADDS, which enable the select activation or suppression of defined subsets of neurons (25–27) as well as targeted CRISPR gene editing (28), is a total game changer—at last we can identify the specific function of particular sets of neurons. Furthermore, our ability to dynamically capture the activity profiles of defined neuronal populations and establish their relationships to behavioral reactions that reflect pain in awake behaving mice is another profound technical breakthrough (29, 30), as is the use of machine learning to capture the data, and artificial intelligence to process and model it (31, 32). Single cell profiling of neurons and non-neuronal cells provides information on the precise nature/identity of all the relevant cells, and if and how they change in disease states and alter their excitability (33–35), while serial EM connectomics is beginning to reveal the precise circuit arrangements in the CNS, and its structural plasticity (36), and now needs to be applied to the spinal cord. Essentially, the black box approach to spinal cord function is almost over, we can literally now begin to take it apart and define exactly how it works and relate this to human/patient studies with functional imaging and other similar measures (37). These are very active areas of interest of many different laboratories.

## Discussion

The purpose of this perspective has been to both highlight the historical importance of the Gate Control theory and assert that it is time to move on to a more up-to-date model, one that is based on our current understanding of spinal cord processing, which is much more than a simple presynaptic gain control, something Pat well recognized in 1996 (38). I predict that a new spinal sensory processing model will emerge soon from the rapidly accumulating new data, that hopefully will be as impactful now as the original model was then, providing us key insight into the operational organization of the spinal cord for physiological nociceptive pain and during pathological pain conditions and again defining potential novel interventional strategies.

I had the privilege of working with Pat Wall at the start of my career and I know he would be as excited as I am at the prospect of a data-driven detailed understanding of the actual processing of sensory information in the spinal cord that drives pain (38), something that is, hopefully, at last imminent. There is no doubt such a new model will still focus on the processing that can lead to or suppress pain, even if the mechanisms and details may differ substantially from the model that has guided us for so many years. However, while now perhaps obsolete, that model was most certainly the major trigger for our ever-improving understanding of spinal sensory processing.

## Author contributions

The author confirms being the sole contributor of this work and has approved it for publication.

## Conflict of interest

The author declares that the research was conducted in the absence of any commercial or financial relationships that could be construed as a potential conflict of interest.

## Publisher's note

All claims expressed in this article are solely those of the authors and do not necessarily represent those of their affiliated organizations, or those of the publisher, the editors and the reviewers. Any product that may be evaluated in this article, or claim that may be made by its manufacturer, is not guaranteed or endorsed by the publisher.

## References

[1] Melzack R Wall PD. On the nature of cutaneous sensory mechanisms. Brain. (1962) 85:331–56. 10.1093/brain/85.2.331 14472486

[2] Mendell LM. Constructing and deconstructing the gate theory of pain. Pain. (2014) 155:210–6. 10.1016/j.pain.2013.12.010 24334188 PMC4009371

[3] Braz J Solorzano C Wang X Basbaum AI. Transmitting pain and itch messages: a contemporary view of the spinal cord circuits that generate gate control. Neuron. (2014) 82:522–36. 10.1016/j.neuron.2014.01.018 24811377 PMC4492533

[4] Zeilhofer HU Ralvenius WT Acuna MA. Restoring the spinal pain gate: GABA(A) receptors as targets for novel analgesics. Adv Pharmacol. (2015) 73:71–96. 10.1016/bs.apha.2014.11.007 25637438

[5] Foster E Wildner H Tudeau L Haueter S Ralvenius WT Jegen M. Targeted ablation, silencing, and activation establish glycinergic dorsal horn neurons as key components of a spinal gate for pain and itch. Neuron. (2015) 85:1289–304. 10.1016/j.neuron.2015.02.028 25789756 PMC4372258

[6] Treede RD. Gain control mechanisms in the nociceptive system. Pain. (2016) 157:1199–204. 10.1097/j.pain.0000000000000499 26817644

[7] Zheng Y Liu P Bai L Trimmer JS Bean BP Ginty DD. Deep sequencing of somatosensory neurons reveals molecular determinants of intrinsic physiological properties. Neuron. (2019) 103:598–616 e7. 10.1016/j.neuron.2019.05.039 31248728 PMC6706313

[8] Gracheva EO Bagriantsev SN. Sensational channels. Cell. (2021) 184:6213–6. 10.1016/j.cell.2021.11.034 34942094

[9] Cathenaut L Leonardon B Kuster R Inquimbert P Schlichter R Hugel S. Inhibitory interneurons with differential plasticities at their connections tune excitatory-inhibitory balance in the spinal nociceptive system. Pain. (2022) 163:e675–88. 10.1097/j.pain.0000000000002460 34490851

[10] Ma L Yu L Jiang BC Wang J Guo X Huang Y. ZNF382 controls mouse neuropathic pain *via* silencer-based epigenetic inhibition of Cxcl13 in DRG neurons. J Exp Med. (2021) 218:920. 10.1084/jem.20210920 34762123 PMC8590274

[11] Dedek A Xu J Lorenzo LE Godin AG Kandegedara CM Glavina G. Sexual dimorphism in a neuronal mechanism of spinal hyperexcitability across rodent and human models of pathological pain. Brain. (2022) 145:1124–38. 10.1093/brain/awab408 35323848 PMC9050559

[12] San Martin VP Sazo A Utreras E Moraga-Cid G Yevenes GE. Glycine receptor subtypes and their roles in nociception and chronic pain. Front Mol Neurosci. (2022) 15:848642. 10.3389/fnmol.2022.848642 35401105 PMC8984470

[13] Xu Q Ford NC He S Huang Q Anderson M Chen Z. Astrocytes contribute to pain gating in the spinal cord. Sci Adv. (2021) 7:eabi6287. 10.1126/sciadv.abi6287 34730998 PMC8565904

[14] Cedeno DL Kelley CA Chakravarthy K Vallejo R. Modulation of glia-mediated processes by spinal cord stimulation in animal models of neuropathic pain. Front Pain Res. (2021) 2:702906. 10.3389/fpain.2021.702906 35295479 PMC8915735

[15] Vallejo R Gupta A Cedeno DL Vallejo A Smith WJ Thomas SM. Clinical effectiveness and mechanism of action of spinal cord stimulation for treating chronic low back and lower extremity pain: a systematic review. Curr Pain Headache Rep. (2020) 24:70. 10.1007/s11916-020-00907-2 32997170

[16] Sdrulla AD Guan Y Raja SN. Spinal cord stimulation: clinical efficacy and potential mechanisms. Pain Pract. (2018) 18:1048–67. 10.1111/papr.12692 29526043 PMC6391880

[17] Linderoth B Foreman RD. Conventional and novel spinal stimulation algorithms: hypothetical mechanisms of action and comments on outcomes. Neuromodulation. (2017) 20:525–33. 10.1111/ner.12624 28568898

[18] Gozani SN. Remote analgesic effects of conventional transcutaneous electrical nerve stimulation: a scientific and clinical review with a focus on chronic pain. J Pain Res. (2019) 12:3185–201. 10.2147/JPR.S226600 31819603 PMC6885653

[19] Middleton SJ Barry AM Comini M Li Y Ray PR Shiers S. Studying human nociceptors: from fundamentals to clinic. Brain. (2021) 144:1312–35. 10.1093/brain/awab048 34128530 PMC8219361

[20] Gradwell MA Boyle KA Browne TJ Bell AM Leonardo J Peralta Reyes FS. Diversity of inhibitory and excitatory parvalbumin interneuron circuits in the dorsal horn. Pain. (2022) 163:e432–52. 10.1097/j.pain.0000000000002422 34326298 PMC8832545

[21] Warwick C Cassidy C Hachisuka J Wright MC Baumbauer KM Adelman PC. MrgprdCre lineage neurons mediate optogenetic allodynia through an emergent polysynaptic circuit. Pain. (2021) 162:2120–31. 10.1097/j.pain.0000000000002227 34130311 PMC8206522

[22] Loken LS Braz JM Etlin A Sadeghi M Bernstein M Jewell M. Contribution of dorsal horn CGRP-expressing interneurons to mechanical sensitivity. Elife. (2021) 10:59751. 10.7554/eLife.59751 34061020 PMC8245130

[23] Choi S Hachisuka J Brett MA Magee AR Omori Y Iqbal NU. Parallel ascending spinal pathways for affective touch and pain. Nature. (2020) 587:258–63. 10.1038/s41586-020-2860-1 33116307 PMC7666110

[24] Arcourt A Gorham L Dhandapani R Prato V Taberner FJ Wende H. Touch receptor-derived sensory information alleviates acute pain signaling and fine-tunes nociceptive reflex coordination. Neuron. (2017) 93:179–93. 10.1016/j.neuron.2016.11.027 27989460

[25] Nagai Y Miyakawa N Takuwa H Hori Y Oyama K Ji B. Deschloroclozapine, a potent and selective chemogenetic actuator enables rapid neuronal and behavioral modulations in mice and monkeys. Nat Neurosci. (2020) 23:1157–67. 10.1038/s41593-020-0661-3 32632286

[26] Roth BL. How structure informs and transforms chemogenetics. Curr Opin Struct Biol. (2019) 57:9–16. 10.1016/j.sbi.2019.01.016 30818201

[27] Barik A Thompson JH Seltzer M Ghitani N Chesler AT. A brainstem-spinal circuit controlling nocifensive behavior. Neuron. (2018) 100:1491–503 e3. 10.1016/j.neuron.2018.10.037 30449655

[28] Meszar Z Kokai E Varga R Ducza L Papp T Beresova M. CRISPR/Cas9-Based mutagenesis of histone H3.1 in spinal dynorphinergic neurons attenuates thermal sensitivity in mice. Int J Mol Sci. (2022) 23:178. 10.3390/ijms23063178 35328599 PMC8955318

[29] Brown EV Malik AF Moese ER McElroy AF Lepore AC. Differential activation of pain circuitry neuron populations in a mouse model of spinal cord injury-induced neuropathic pain. J Neurosci. (2022) 42:3271–89. 10.1523/JNEUROSCI.1596-21.2022 35256528 PMC8994550

[30] Sullivan SJ Sdrulla AD. Excitatory and inhibitory neurons of the spinal cord superficial dorsal horn diverge in their somatosensory responses and plasticity *in vivo*. J Neurosci. (2022) 42:1958–73. 10.1523/JNEUROSCI.1860-21.2021 35046121 PMC8916760

[31] Zhang Z Roberson DP Kotoda M Boivin B Bohnslav JP Gonzalez-Cano R. Automated preclinical detection of mechanical pain hypersensitivity and analgesia. Pain. (2022). 10.1097/j.pain.0000000000002680. [Epub ahead of print]. 35543646 PMC9649838

[32] Medlock L Sekiguchi K Hong S Dura-Bernal S Lytton WW Prescott SA. Multiscale computer model of the spinal dorsal horn reveals changes in network processing associated with chronic pain. J Neurosci. (2022) 42:3133–49. 10.1523/JNEUROSCI.1199-21.2022 35232767 PMC8996343

[33] Clerc N Moqrich A. Diverse roles and modulations of IA in spinal cord pain circuits. Cell Rep. (2022) 38:110588. 10.1016/j.celrep.2022.110588 35354022

[34] Stachowski NJ Dougherty KJ. Spinal inhibitory interneurons: gatekeepers of sensorimotor pathways. Int J Mol Sci. (2021) 22:667. 10.3390/ijms22052667 33800863 PMC7961554

[35] Renthal W Tochitsky I Yang L Cheng YC Li E Kawaguchi R. Transcriptional reprogramming of distinct peripheral sensory neuron subtypes after axonal injury. Neuron. (2020) 108:128–44.e9. 10.1016/j.neuron.2020.07.026 32810432 PMC7590250

[36] Witvliet D Mulcahy B Mitchell JK Meirovitch Y Berger DR Wu Y. Connectomes across development reveal principles of brain maturation. Nature. (2021) 596:257–61. 10.1038/s41586-021-03778-8 34349261 PMC8756380

[37] Irimia JD. Van Horn, Mapping the rest of the human connectome: atlasing the spinal cord and peripheral nervous system. Neuroimage. (2021) 225:117478. 10.1016/j.neuroimage.2020.117478 33160086 PMC8485987

[38] Wall PD. Comments after 30 years of the gate control theory. Pain Forum. (1996) 5:12–22. 10.1016/S1082-3174(96)80063-8

